# Assessing the interrelationship between stigma, social influence, and cervical cancer prevention in an urban underserved setting: An exploratory study

**DOI:** 10.1371/journal.pone.0278538

**Published:** 2022-12-01

**Authors:** Caryn E. Peterson, J. Andrew Dykens, Stevan M. Weine, Hunter K. Holt, Judes Fleurimont, Christina G. Hutten, John Wieser, Fida Abuisneineh, Saria Awadalla, Natalia P. Ongtengco, Nicole Gastala, Ian G. Jasenof

**Affiliations:** 1 Division of Epidemiology and Biostatistics, School of Public Health, University of Illinois at Chicago, Chicago, Illinois, United States of America; 2 College of Medicine, University of Illinois at Chicago, Chicago, Illinois, United States of America; 3 Mile Square Health Center, UI Health, Chicago, Illinois, United States of America; 4 University of Illinois at Chicago, Chicago, Illinois, United States of America; CUHK: The Chinese University of Hong Kong, HONG KONG

## Abstract

In the US, incidence and mortality from cervical cancer disproportionately affects racial/ethnic minorities and low-income women. Despite affordable access to primary and secondary prevention measures at Federally Qualified Health Centers (FQHCs), Human Papillomavirus (HPV) vaccination and screening rates are low, suggesting the presence of non-financial barriers to uptake in this population. This explanatory sequential mixed-methods study sought to explore factors that influence the acceptability of cervical cancer prevention services among parents and legal guardians of vaccine-eligible girls attending an urban FQHC and to assess social influences related to cervical cancer prevention. Participants included eight mothers, one father, and two grandparents/legal guardians. Nine participants self-identified as Black/Afro-Caribbean, or African American, two as Latinx, and one as Native American. The quantitative data suggested discordance between participants’ cervical cancer prevention knowledge and their practices. Most indicated that their daughters had received the HPV vaccine but were unsure about HPV transmission modes. Qualitative data revealed that participants were comfortable disclosing information on HPV infection and vaccination status, and most women were likely to share information related to cervical cancer testing and diagnosis. Few comments indicated personal stigma on the part of participants, but there was frequent expression of perceived public stigma (shaming and blaming women), gender differences (men are indifferent to risk), and distrust of the healthcare system. Findings highlight several concepts including the disharmony between knowledge and practice, prevalent perceived public stigma, cumbersome attitudes on the part of men regarding HPV and cervical cancer, and distrust of the healthcare system.

## Introduction

Cervical cancer is the second-leading cause of cancer deaths among U.S. women aged 20–39 years and disproportionately affects racial and ethnic minorities and low-income women [1]. Vaccination against human papillomavirus (HPV) and cervical screening are highly effective prevention strategies for cervical cancer [2]; yet despite these proven interventions, both HPV vaccination [3] and screening rates [4] fall well below the Healthy People 2020 targets. Access and affordability issues have long been drivers of suboptimal utilization of health prevention services, including screening and vaccinations [5]. Federally Qualified Health Centers (FQHCs) can play a critical role in increasing vaccination and screening rates in uninsured and underinsured populations. However, uptake is low in these populations [6–8]. FQHC patients have access to programs that eliminate the cost of vaccination (i.e., the US federal government’s Vaccines for Children Program) and mandated coverage for cervical cancer screening (i.e., the Affordable Care Act), suggesting the presence of other barriers to cancer prevention beyond access and affordability.

Stigma is a potent negative social influencer and is initiated and perpetuated by social structures and power dynamics that ascribe undesirable characteristics to particular conditions [9, 10]. Social stigma can manifest as perceived public stigma (i.e., how one thinks others view and treat them) and personal stigma (i.e., how one actually views and treats others themselves) [11]. In addition, the expression of attitudes related to stigma is moderated by social influence—that is by the ability of individuals to affect one another’s thoughts, ideas, and behaviors [12, 13]. In these ways, stigma may directly and indirectly contribute to vaccine and screening hesitancy [14]. For example, stigmas around sexual behavior and HPV infection [15] may contribute to vaccine and screening hesitancy by causing individuals to avoid engagement with providers [16]. Stigma associated with HPV infection and cervical cancer may lead to fears related to disclosure and diagnosis [17], which in turn may cause individuals to avoid care engagement and screening [18, 19]. Our recent scoping review used Stangl and colleagues’ Health Stigma and Discrimination Framework [20] to determine stigmas related to HPV infection and vaccination, as well as cervical cancer and screening in the US. Briefly, fear of social judgement and rejection, self-blame, and shame were found to be drivers of stigma. Social norms that provided motivation to receive HPV vaccination and screening served as positive facilitators. Gender and social norms were negative facilitators of stigma [14]. Understanding how these factors contribute to the acceptability and adoption of cervical cancer prevention strategies is critical to preventing cervical cancer. The objectives of this exploratory study were to explore the role that social influences in general, and stigma in particular, play in influencing the acceptability of cervical cancer prevention services among parents and legal guardians of vaccine-eligible girls attending an urban FQHC clinic, to assess the social influences related to HPV vaccination and cervical cancer screening, and to evaluate the feasibility of engaging study participants in questions relating to these factors.

## Materials and methods

### Setting & study design

This exploratory study took place at the Mile Square Health Center-Main (MSHCM) clinic in the city of Chicago. Affiliated with the University of Illinois at Chicago Hospital Health Sciences System, MSHCs are a group of 14 FQHCs serving more than 40,000 patients annually. We utilized an explanatory sequential (Quan+Qual) mixed-methods study design to identify potential trends in the data and to focus on the lived experiences of these study participants [21–23]. Quantitative data were collected using a self-administered questionnaire to gather information on participants’ demographics, assess their knowledge, practices, and attitudes related to cervical cancer prevention. The questionnaire included closed-ended, quantitative questions seeking information on participant’s perceptions of cancer stigma, opinions, and attitudes. We included questions from the Cancer Stigma Scale adapted to cervical cancer screening and HPV [24]. Questionnaires were field tested for comprehension prior to the initiation of the study. Following the completion of the quantitative portion of the study, qualitative data were collected through semi-structured interviews lasting between 15 and 30 minutes. Participants’ responses related to knowledge and practices derived from the quantitative survey were further explored in interview questions related to HPV and cervical cancer in their daily lives, with a focus on their own experiences as well as their perceptions of members of their social networks. Furthermore, responses related to attitudes revealed in the quantitative survey were explored in interview questions examining positive and negative social influences impacting cervical cancer prevention, with a particular focus on perceived public stigma and personal stigma. The interview guide was reviewed by the Mile Square Health Centers (MSHC) staff and the MSHC Research Council. This study was approved by the University of Illinois at Chicago (UIC) Institutional Review Board (IRB) and the MSHC Research Council.

#### Eligibility & recruitment

Study participants were parents (mothers or fathers) or legal guardians of HPV vaccine-eligible girls (i.e., assigned female gender at birth and ages 9–17 years) attending the MSHCM pediatric clinic. Study staff identified eligible participants from the clinic’s weekly schedule of incoming patients, and potential participants were contacted by phone and invited to participate in the study. Following completion of the informed consent process, participants received a secure electronic link to the self-administered survey using REDCap software. Study staff reviewed each participant’s completed survey and then scheduled a telephone interview. Semi-structured interviews were audio recorded and later transcribed verbatim. Participants received a $50 gift card. This incentive was approved by the IRB as adequate for the participants’ time without being coercive.

#### Statistical analysis

Characteristics of the study population as well as knowledge, practices, and attitudes related to cervical cancer prevention were tabulated. Bivariate analysis was conducted to explore differences in the distribution of attitudes by women’s screening time (< 3 years vs. ≥ 3 years) to align with U.S. Preventive Services Task Force cervical cancer screening recommendations [25] and child’s vaccination status (vaccinated vs. unvaccinated) using Fisher’s Exact test statistics. Quantitative analysis was conducted in SAS 9.4 (Carey, NC).

For the qualitative data analysis, we used an inductive, iterative approach [26] using Dedoose software [27]. Recorded interviews were transcribed, data were entered into Dedoose, and we proceeded with a stepwise analysis. The data set was divided between two research team members (JAD and SMW), and from line-by-line reading, initial overarching concepts were identified. We developed the coding scheme as we labeled, defined, refined, and grouped concepts into categories corresponding to the initial conceptual framework focusing on perceived public stigma (i.e., how one thinks others would view and treat them) and personal stigma (i.e., how one actually would view and treat others themselves) [11, 28]. Next, a codebook was developed consisting of the labels, definitions, and illustrations of formulated categories. Reviewers concurred on the codebook, which we piloted and revised using selections of text from the data. We coded the entire dataset using the coding scheme by first reviewing the interview transcripts and field notes and assigning sections of text to the corresponding coding categories. We examined the relationship within and between codes to identify overarching themes and patterns of variation. We continued pattern coding until saturation [29]. At each of the aforementioned steps we wrote short, descriptive memos to a) document ideas during preliminary data review, b) define codes during coding, c) concisely describe items not coded, and d) describe the outcomes of specific Dedoose queries. We then scanned memos and sorted them to form clusters, categories, and causal networks [30].

## Results

### Study population

The non-probabilistic sample included 11 participants—eight mothers, one father, and two grandparents/legal guardians. The mean age of participants was 43.1 years (SD = 9.4), and all women in the sample were eligible for cervical cancer screening. Racial/ethnic heritage were not mutually exclusive categories, and nine participants self-identified as Black/Afro-Caribbean, or African American, two as Latinx or Hispanic American, and one as Native American. More than half (55%) of participants had graduated from high school or received their GED certificate, and 45% had graduated from college or completed some college. The majority (64%) of participants were covered by Medicaid, three (27%) were covered by private insurance or a Marketplace Plan, and one had no health insurance. The majority (82%) of participants’ children were covered by Medicaid (Table 1).

**Table 1:** Participant characteristics.

CHARACTERISTIC	N = 11 (%)
Participant Eligibility	
Mother	8 (72.7)
Father	1 (9.1)
Grandparent/Legal guardian	2 (18.2)
Age, mean years [SD]	43.1 (9.4)
Age, range years	33–62
Race/Ethnic Heritage ^a^ (check all that apply)	
Black, Afro-Caribbean, or African American	9 (81.8)
Latinx or Hispanic American	2 (18.1)
Native American or Alaskan Native	1 (9.1)
Educational Attainment	
High School or GED	6 (54.5)
Some College	1 (9.1)
Graduated from College	4 (36.4)
Marital Status	
Married	3 (27.3)
Separated	1 (9.1)
Divorced	1 (9.1)
Single, never married	6 (54.5)
Participants’ Insurance	
No coverage	1 (9.1)
Medicaid	7 (63.6)
Private insurance such as CIGNA	2 (18.2)
Marketplace Plan such as Blue Choice	1 (9.1)
Child’s Insurance	
No coverage	1 (9.1)
Medicaid	9 (81.8)
Missing	1 (9.1)

^a^ Categories not mutually exclusive.

#### Knowledge, practices, and attitudes related to cervical cancer prevention

The majority of participants reported that they have heard about and understand HPV (64%), cervical cancer (91%), the HPV vaccine (73%) and Pap test (100%). Most reported that their daughters had received the HPV vaccine (73%). However, there was some inconsistency between this practice and some aspects of participants’ knowledge. For example, only 27% of participants felt they understood how to protect their child from HPV very well and only 36% felt they understood the risks associated with HPV very well. Most participants were unsure how HPV is transmitted (55%) and whether it was likely that a diagnosis of HPV meant one could infect someone else (45%).

Of the 10 women in the study, all were eligible for cervical cancer screening. Although eight women reported having had a Pap test, three reported receiving a test more than three years ago. There was some discordance between participants’ screening practices and their knowledge. Four of the eight women reporting past Pap tests felt that they understood how to protect themselves from cervical cancer, while two felt they did not and two were unsure (Table 2).

**Table 2:** Practices and knowledge related to cervical cancer prevention.

PARTICIPANT RESPONSES	N = 11 (%)
**HPV**	
*Did your daughter ever receive the HPV vaccine shot/the shot that prevents infection with HPV*	
Yes	8 (72.7)
No	3 (27.3)
*Have you heard about HPV*, *also called the Human Papillomavirus*?	
I have heard about HPV and understand what it is	7 (63.6)
I have heard of HPV, but I’m not sure I understand what it is	3 (27.3)
I have not heard of HPV	1 (9.1)
*How well do you understand the risks associated with HPV*?	
Very well	4 (36.4)
Somewhat well	4 (36.4)
I do not understand the risks at all	3 (27.2)
*How well do you think your close friends and family understand the risks associated with HPV*?	
Very well	0 (0.0)
Somewhat well	7 (63.6)
They not understand the risks at all	4 (36.4)
*Have you heard about a vaccine that prevents HPV infection called the HPV vaccine*?	
I have heard about the HPV vaccine and understand what it is	8 (72.7)
I have heard of HPV, but I’m not sure I understand what it is	1 (9.1)
I have not heard of the HPV vaccine	2 (18.2)
*How well do you understand how to protect your child from HPV*?	
Very well	3 (27.3)
Somewhat well	6 (54.5)
Unsure	2 (18.2)
*How likely do you think it is that a person can get HPV from someone else who has HPV*?	
Likely	5 (45.5)
Unlikely	0 (0.0)
Unsure	6 (54.5)
*If you were diagnosed with HPV*, *how likely is it that you would infect someone else*?	
Likely	4 (36.4)
Unlikely	2 (18.2)
Unsure	5 (45.4)
**Cervical Cancer**	
*Have you heard about cervical cancer*?	
I have heard about cervical cancer and understand what it is	10 (90.9)
I have heard about cervical cancer but I’m not sure I understand what it is	1 (9.1)
I have not heard of about cervical cancer	0 (0.0)
*Have you heard about the Pap test (also called Pap Smear) that checks to see if you have cervical cancer*?	
I have heard about the Pap test and understand what it is	11 (100.0)
I have heard about the Pap test, but I’m not sure I understand what it is	0 (0.0)
I have not heard about the Pap test	0 (0.0)
*If a woman had a positive Pap test for cervical cancer*, *how likely is it that she could be cured*?	
Likely	7 (63.6)
Unlikely	0 (0.0)
Unsure	4 (36.4)
*If a woman were diagnosed with cervical cancer*, *how likely is it that she could be cured*?	
Likely	4 (36.4)
Unlikely	0 (0.0)
Unsure	7 (63.6)
**MOTHERS’/WOMENS’ RESPONSES (n = 10)**	**N = 10 (%)**
*Have you had a Pap test*?	
Yes	8 (80.0)
No	2 (20.0)
I don’t recall	0 (0.0)
*When was the last time you had a Pap test*?	
Within the past 12 months	3 (30.0)
More than 1 year ago	1 (10.0)
More than 2 years ago	1 (10.0)
More than 3 years ago	3 (30.0)
I don’t recall	0 (0.0)
I have not received a Pap test	2 (20.0)
*Do you feel that you understand how to protect yourself from cervical cancer*?	
Yes	4 (40.0)
No	2 (20.0)
Unsure	2 (20.0)
Missing	2 (20.0)

Most participants indicated that they would be likely to disclose information regarding their own (or their child’s) HPV status, as well as their child’s vaccination status. Similarly, most women were likely to share information related to cervical cancer testing, receipt of a positive Pap test, and a cervical cancer diagnosis with healthcare providers, a person close to them, and future sexual partners (Table 3). When asked if they were to receive an abnormal Pap test, 90% of women indicated that they would return for an in-office colposcopy. However, women described several barriers to follow up including, cost, fear, inability to take time off work, lack of childcare, a desire to avoid a bad result, and a belief that nothing could be done about their diagnosis. In the bivariate analysis assessing differences in attitudes related to cervical cancer there were no statistically significant differences by women’s screening time. For example, women aligned with USPSTP recommendations (i.e., screened within the past three years) were just as likely to disclose a potential cervical cancer diagnosis or positive Pap test as women screened three or more years ago. Similarly, there were no statistically significant differences in participants’ attitudes by child’s HPV vaccination status (Data not shown).

**Table 3:** Disclosure related to cervical cancer prevention HPV.

ALL PARTICIPANT RESPONSES	N = 11 (%)
*If you decide to* *vaccinate your child* *against HPV infection*, *would you choose to let people or a person close to you know*?	
Yes	8 (72.7)
No	3 (27.3)
*If your* *child was diagnosed* *with HPV*, *would you share that information with your own healthcare provider*?	
Likely	8 (72.7)
Unlikely	0 (0.0)
Unsure	3 (27.3)
*If your child were diagnosed with HPV*, *would you share that information with a person close to you*?	
Likely	8 (72.7)
Unlikely	1 (9.1)
Unsure	2 (18.2)
*If you were diagnosed with HPV*, *would you share this information with your healthcare provider*?	
Likely	8 (72.7)
Unlikely	0 (0.0)
Unsure	3 (27.3)
*If you were diagnosed with HPV*, *would you share this information with a person close to you*?	
Likely	8 (72.7)
Unlikely	1 (9.1)
Unsure	2 (18.2)
*If you were diagnosed with HPV*, *would you share this information with future sexual partners*?	
Likely	9 (81.8)
Unlikely	0 (0.0)
Unsure	2 (18.2)
**MOTHERS’/WOMENS’ RESPONSES**	**N = 10 (%)**
*If you decide to get* *tested* *for cervical cancer*, *would you let people or a person close to you know*?	
Yes	6 (60.0)
No	2 (20.0)
Missing	2 (20.0)
*If you were* *diagnosed* *with cervical cancer*, *would you share this information with healthcare providers*?	
Likely	7 (70.0)
Unlikely	0 (0.0)
Unsure	1 (10.0)
Missing	2 (20.0)
*If you were diagnosed with cervical cancer*, *would you share this information with a person close to you*?	
Likely	7 (70.0)
Unlikely	0 (0.0)
Unsure	1 (10.0)
Missing	2 (20.0)
*If you were diagnosed with cervical cancer*, *would you share this information with future sexual partners*?	
Likely	8 (80.0)
Unlikely	0 (0.0)
Unsure	0 (0.0)
Missing	2 (20.0)
*If you were diagnosed with a positive Pap test*, *would you share this information with healthcare providers*?	
Likely	7 (70.0)
Unlikely	0 (0.0)
Unsure	1 (10.0)
Missing	2 (20.0)
*If you were diagnosed with a positive Pap test*, *would you share this information with a person close to you*?	
Likely	7 (70.0)
Unlikely	0 (0.0)
Unsure	1 (10.0)
Missing	2 (20.0)
*If you were diagnosed with a positive Pap test*, *would you share this information with future sexual partners*?	
Likely	6 (60.0)
Unlikely	1 (10.0)
Unsure	0 (0.0)
Missing	3 (30.0)

**Social influences related to HPV and cervical cancer.** Study participants tended to disagree with statements indicating discomfort with someone infected with HPV (45%) or with cervical cancer (73%). This pattern persisted with respect to avoidance and blame. Participants disagreed with statements that they would avoid someone with HPV (91%) or cervical cancer (100%). Similarly, participants did not fault others for their HPV infection (82%) or for developing cervical cancer (91%). However, when asked how likely it is for others within their social network to blame a person for their HPV infection, only 27% believed it was unlikely (Table 4). There were no statistically significant differences in participants’ attitudes by child’s HPV vaccination status, but responses suggest that participants who vaccinated their daughters were less likely to stigmatize HPV. Compared to participants whose daughters were unvaccinated, a greater proportion of participants with vaccinated daughters disagreed with the following statements: “HPV usually ruins close personal relationships”, “I would try to avoid someone with HPV”, If a person has HPV, it’s probably their fault”, and “A person with HPV is responsible for getting this condition” (Data not shown).

**Table 4:** Stigma related to cervical cancer prevention HPV.

ALL PARTICIPANT RESPONSES	N = 11 (%)
*I would feel uncomfortable around someone who has HPV*	
Agree	4 (36.4)
Disagree	5 (45.4)
Unsure	2 (18.2)
*I would try to avoid someone with HPV in my day-to-day life*	
Agree	1 (9.1)
Disagree	10 (90.9)
*I would find it hard to talk to someone who has HPV*	
Agree	0 (0.0)
Disagree	11 (100.0)
Unsure	0 (0.0)
*HPV usually ruins close personal relationships*	
Agree	3 (27.3)
Disagree	8 (72.7)
*Once you’ve had HPV you can never be totally cured of it*	
Agree	5 (45.5)
Disagree	6 (54.5)
*If someone were diagnosed with HPV*, *how likely is it that people would blame that person*?	
Likely	5 (45.4)
Unlikely	3 (27.3)
Unsure	3 (27.3)
*If you were diagnosed with HPV*, *how likely is it that you would blame yourself*?	
Likely	2 (18.2)
Unlikely	5 (45.4)
Unsure	4 (36.4)
*If a person has HPV*, *it’s probably their fault*	
Agree	2 (18.2)
Disagree	9 (81.8)
*A person with HPV is responsible for getting for this condition*	
Agree	3 (27.3)
Disagree	8 (72.7)
*I would feel uncomfortable around someone who has cervical cancer*	
Agree	3 (27.3)
Disagree	8 (72.7)
Unsure	0 (0.0)
*I would try to avoid someone with cervical cancer in my day-to-day life*	
Agree	0 (0.0)
Disagree	11 (100.0)
*I would find it hard to talk to someone who has cervical cancer*	
Agree	0 (0.0)
Disagree	10 (90.9)
Unsure	0 (0.0)
Missing	1 (9.1)
*Cervical cancer usually ruins close personal relationships*	
Agree	2 (18.2)
Disagree	9 (81.8)
*Once you’ve had cervical cancer you can never be totally cured of it*	
Agree	3 (27.3)
Disagree	8 (72.7)
*If a woman has cervical cancer*, *it’s probably her fault*	
Agree	0 (0.0)
Disagree	10 (90.9)
Missing	1 (9.1)
*A woman with cervical cancer is responsible for getting this condition*	
Agree	1 (9.1)
Disagree	10 (90.9)

#### Qualitative analysis

The quantitative data suggested that participants themselves did not stigmatize HPV infection or a diagnosis of cervical cancer. However, they perceived that people close to them held different views. This apparent difference between participants’ perceptions and those of their social network was further explored and expanded upon in guided interviews. Semi-structured interviews sought to further explore participants’ experiences of HPV and cervical cancer in their daily lives, as well as their perceptions of the attitudes of members of their social networks with respect to HPV infection and vaccination and cervical cancer and screening. The qualitative analysis of these data reinforced and expanded upon these findings through four main themes related to social influences: perceived public stigma (Shaming and blaming women), gender differences (Men are indifferent to risk), institutional perception (distrust of the healthcare system), and positive social influence (Influencing others through shared experiences).

*Perceived public stigma*. Although participants seldom described experiences or feelings of personal stigma, there were numerous expressions of perceived public stigma related to HPV infection and cervical cancer diagnosis. Participants’ shared perceptions that members of their social networks blame women for their cervical cancer diagnosis. Furthermore, there is a sense within their communities that HPV infection is viewed as shameful. Participants also believed that men within their social networks do not acknowledge their role in a female partner’s HPV infection.

#### Shaming and blaming women

Participants stated that people do not openly talk about HPV infection because there is shame associated with it. “*Because it is something you live with the rest of your life*, *and it can be passed on or whatever*. *If you get it*, *the disclosure*, *having to disclose it with your partner*, *and the effects that it can take on a relationship if you had it*, *and all of that*.” (part. #5, mother) Many participants also commented on “*blame*” toward women who have cervical cancer. Participants stated that others believe that these women must have "*done something*"(part. #10, mother) to end up with this diagnosis. “*They blame the women*.” (part. #5, mother) It was stated that it “*is the woman that gave it to the man*.” (part. #8, mother) Another participant indicated blame in commenting that many people feel that there is a behavior that causes cervical cancer. It was stated that to avoid getting cervical cancer, you have to "*keep your legs closed*."(part. #9, grandmother) It was also stated that, “*In letting that person with the cervical cancer isolate themselves from them or distance themselves from them*, *because in their minds it’s maybe that it’s contagious*.” (part. #4, mother) Regarding the perceptions of others, one participant expressed that there is a thought that "*more conservative*"(part. #4, mother) women (those with fewer sexual partners) were more judgmental. “*I think it’s just because it’s cancer*. *When people hear the word cancer*, *they get nervous*, *they get cautious*. *They think is it contagious*. *They’re unaware*, *so they’re like*, *‘We don’t know what’s going to happen*.*’ So they don’t want to come around*.” (part. #10, mother) Participants further illustrated blame in stating that women who have cervical cancer or are HPV positive are “*spoiled*.” (part. #4, mother) “*Yep*. *They nasty*, *or they sleep around*, *or they wild*, *or they out there not protecting themselves and not being cautious and just sleeping around*.” (part. #5, mother)

#### Gender differences

*Men are indifferent to risk*. Participants expressed that men that they know do not take precautions against HPV and that men’s behavior is riskier than women’s. One participant stated that, “*a lot of men can be degrading*. *A lot of men don’t care if they do have it*. *They will continue if it’s contagious or spreadable*. *Some men that I have known wouldn’t care*. *They would just spread it if they can or they would just not take it as serious as doctors*, *and women would take it seriously*.” (part. #2, father) In addition, this participant noted, “*A woman that has cervical cancer*, *I believe that some men in my family members don’t take it seriously*. *They may have feelings about the situation but if it don’t concern them*, *they’re not interested*. *Or they’ll have less pity for them unless it’s their mother or a spouse*.” (part. #2, father) It is perceived that men judge women differently than other men. *“It can be a double standard when it comes to something like that because [being infected with HPV is] not portrayed as a serious matter as far as it would be for a woman*.*”* (part. #2, father) Another participant remarked that among men there is blaming and shaming of the women who are HPV positive. It was stated that “*they would single out more women than men*” (part. #4, mother) when it comes to HPV infection. Finally, a participant stated, “*I feel that a lot of men don’t have a lot of knowledge towards HPV*, *I think*.” (part. #2, father).

#### Institutional perceptions

*Distrust of the healthcare system*. Participants’ comments indicated that there was a pervasive distrust of the HPV vaccine, recommendations from doctors, and the healthcare system. One participant commented that, “*I know some people don’t want to vaccinate because they feel like the cancer is in the vaccination*.” (part. #6 mother) Another remark was that it was “*Not trustworthy of the doctors*. *A lot of doctors are not good out here*. *Some doctors are in it for the money*. *And some doctors*, *in my way of life*… *by me being African American*, *it’s a stigma towards doctors that won’t treat African Americans as well as another race*. *So our stigma towards doctors are not well*.” (part. #2, father) Finally, it was pointed out that, “*The relationship between doctors and patient in my society is not a great relationship*. *So a lot of times they will say*, *‘Oh*, *your child needs a vaccine*.’ *But then again*, *you will hear negative things about the vaccine*, *maybe horrible side effects within that person*. *And it would discourage them from not wanting to treat that child or their self to get the vaccine*.” (part. #2, father) In addition, a participant discussed that having limited coverage by some insurance plans causes people to distrust the healthcare system. *“Definitely*. *With my 23-year-old*, *she just feels like*… *She has to get her a better job where she can get better insurance because with public aide it’s just giving us the bare minimum insurance*. *She’s going through what I went through with these bacteria infections*. *I feel that a lot people just have lost trust in the system*.*”* (part. #8, mother).

#### Positive social influences

*Influencing others through sharing experiences*. Participants reported that the sharing of personal stories regarding cervical cancer prevention is a good way to influence others to make similar choices. One participant stated, "*I’m open to share it*. *They look at that and they take heed*."(part. #8, mother) It was also stated, “*and then when I got my situation taken care of*, *I went back to work and I started talking about it*, *and it made two other coworkers*… *We’re all from the same neighborhood*. *Made them go and get they self-checked out*.” (part. #8, mother) This same participant remarked that "*I literally talked them into going and seeing about it*"(part. #8, mother) when discussing cervical cancer screening. A further comment was, “*Actually I had a conversation with some of my family members about it and let them know that I did do that*. *And then I found out a lot of them got it too and had it for their young lady children as well*.” (part. #10, mother) Finally a participant stated, “*Because I feel confident about the decision*. *Now it might help another family*, *another parent*. *It may not*, *but still*, *I am going to share*. *Yeah*.” (part. #4, mother)

## Discussion

Our findings from this explanatory sequential mixed-methods study highlight several concepts including the disharmony between practice and knowledge, prevalent perceived public stigma—including dismissive attitudes on the part of men regarding HPV and cervical cancer, distrust of the healthcare system, and the promise related to positive social influence.

The first phase from quantitative data suggested possible discordance between participants’ cervical cancer prevention practices and their knowledge. Although most participants indicated that their daughters had received the HPV vaccine, they were unsure about HPV transmission modes. All the women in the study reported past receipt of a Pap test but not all had received a test within the past three years. We hypothesize that incongruence between practice and knowledge may result in inconsistent prevention behaviors such as guideline concordant screening.

Regarding stigma, the qualitative data reinforced a common quantitative finding. Notably, participants themselves were comfortable disclosing information on HPV infection and vaccination status, and most women were likely to share information related to cervical cancer testing and diagnosis. While there were minimal comments expressing personal stigma on the part of participants, there was frequent expression of perceived public stigma. Participants did not stigmatize HPV and cervical cancer themselves however, this attitude did not extend to their social network—particularly with respect to HPV infection. Women participants’ expression of perceived public stigma indicates a fear of being discriminated against. This revealed that there is a view that others may hold prejudices toward women who are HPV positive or diagnosed with cervical cancer.

The qualitative phase also expanded on the findings from the quantitative phase indicating gender differences, a distrust of the healthcare system, and indications of positive social influence related cervical cancer and HPV vaccination interpersonal communication. These data illustrated that responses from participants indicated differences in the way genders perceive and react to circumstances regarding cervical cancer and cervical cancer prevention. This suggests that there are direct and indirect differences in the way that women and men express their perceptions around HPV infection or a cervical cancer diagnosis, especially related to perceived public stigma, blame, personal responsibility, empathy, valuing prevention, and valuing reliable information. Our findings indicate that men’s attitudes and behavior may be indifferent to HPV risk. In addition, there is a perception that men may blame or shame women for a cervical cancer or HPV diagnosis.

Many participants indicated that there exists a distrust of the healthcare system in their communities which is problematic for positive cervical cancer prevention behaviors. Participants hold a distrust of the HPV vaccine, itself, of the doctors who are recommending the vaccine, and of the healthcare system as a whole. There was an indication that the distrust is born through a generational conditioning. In particular, it is notable that participants stated that African Americans believe that doctors won’t treat African Americans as well as another race. This is consistent with findings from recent studies [31–34] and presents a major barrier to addressing cervical cancer screening and HPV vaccine hesitancy.

The qualitative analysis also illustrated that women often use their own experiences to influence the decisions of others. Participants expressed belief that this type of communication has a positive effect on the behavior of their peers. Individuals who have, themselves, taken action by getting screened for cervical cancer or having their daughter get the HPV vaccine may act as sources of positive social influence by sharing their decisions and experiences with others.

These findings suggest that leveraging positive social influence may be a useful strategy to address identified perceived cervical cancer and HPV-related public stigmas, men’s indifference to HPV risk, and the existing distrust of the medical system. Positive social influence has been shown to influence the behavior of decision-makers [35–38] and may be impactful at the interpersonal level. Sharing information within social circles may be a good way to build knowledge and influence decisions of family members or close contacts relative to HPV and cervical cancer prevention.

## Limitations

This exploratory study begins to address an important gap in the literature defining the relationship between the effect of social influences on the acceptability of cervical cancer prevention and the hesitancy to seek these health services—particularly among those receiving care at FQHCs. Nevertheless, we acknowledge important limitations. The quantitative results are not hypothesis driven and unencumbered by power and sample size limitations however, inference and generalizability are limited and conclusions regarding the causal nature of these relationships cannot be established. Given the small sample size and minimal variation in vaccination and screening outcomes we were unable to examine the relationship between measures of social influence and these outcomes through multivariate models. Our results provide insight into the ways in which stigma, gender differences, and medical mistrust may negatively influence decisions related to cervical cancer prevention and suggest a path of further exploration to elucidate these relationships.

## Conclusions

Our findings illustrated various barriers to cervical cancer prevention with consistency between primary (HPV vaccination) and secondary (cervical cancer screening) prevention as well as perceptions and attitudes regarding HPV infection and cervical cancer diagnoses. Future public health interventions should focus on a unified message to increase the understanding of the relationship between HPV and cervical cancer as well as the various modalities for the prevention of cervical cancer. Efforts should also focus on addressing men’s attitudes, in particular, regarding HPV and cervical cancer. Men’s attitudes as well as women’s perceived attitudes may contribute to our finding of perceived public stigma. More research should be conducted to better understand the relationship between this observable stigma and the practice of HPV vaccination and cervical cancer screening. It is likely that decision makers are discouraged from the uptake of cervical cancer prevention services due to these social factors. Our study also reinforces previous findings of distrust of the healthcare system, which adversely affects uptake. In addressing these critical issues, our findings suggest that interventions leveraging positive social influence may be a promising approach to addressing the identified barriers. In advancing the broader goal of the elimination of cervical cancer as a public health problem, robust research is needed to better understand the social phenomena that are directly related to factors influencing cervical cancer primary and secondary prevention.
